# Exercise may serve as a potential preventive strategy for mitochondria influencing the occurrence of heart failure with preserved ejection fraction

**DOI:** 10.1515/jtim-2026-0027

**Published:** 2026-04-27

**Authors:** Lin Zheng, Zhengkai Wang, Xuezheng Xuan, Yiyao Zhang, Kun Hua, Xiubin Yang

**Affiliations:** Department of Cardiac Surgery, Beijing Anzhen Hospital, Capital Medical University, Beijing, China; Beijing Institute of Heart, Lung and Blood Vessel Diseases, Beijing Anzhen Hospital, Capital Medical University, Beijing, China

## Introduction

Heart failure with preserved ejection fraction (HFpEF) represents a significant and growing global public health challenge. Currently accounting for nearly half of all heart failure cases in high-income countries, HFpEF is characterized by typical symptoms and signs of heart failure in the presence of a left ventricular ejection fraction ≥ 50%.^[1]^ Its rising prevalence is closely linked to population aging and the increasing incidence of metabolic comorbidities such as obesity, hypertension, and diabetes. While the incidence of HFpEF remains high, the treatment options are also very limited. Currently, most drug interventions that are beneficial for heart failure and reduced ejection fraction have a disappointing impact on the HFpEF population. Therefore, new prevention and treatment strategies for HFpEF patients may be the focus of future research.^[2]^ Recent mechanism studies have found that mitochondrial dysfunction is the core pathological mechanism of HFpEF. It may be involved in the development of HFpEF through multiple aspects such as metabolic stress, oxidative damage and cellular energy impairment. This review synthesizes current evidence and proposes that organized exercise training is an effective non-pharmaceutical intervention that can regulate mitochondria, thereby potentially preventing or improving the progression of HFpEF.

## Regular physical activity improves mitochondrial function through multiple complementary pathways

**Figure 1:**
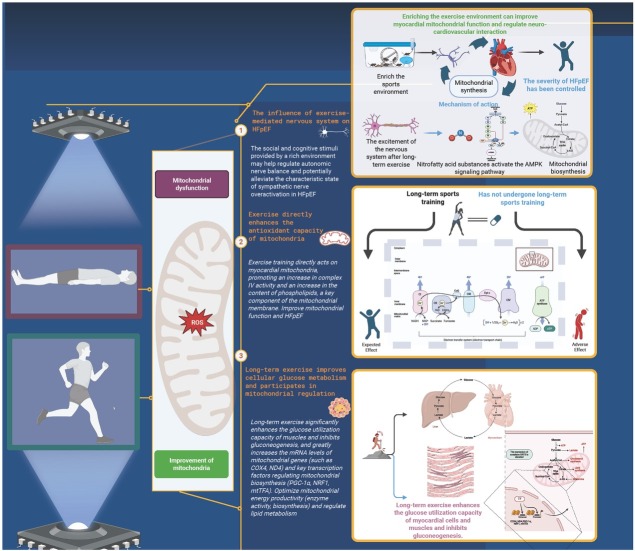
How does mitochondrial dysfunction mediate the production of HFpEF. HFpEF: heart failure with preserved ejection fraction. Image created by BioRender.com.

The pathogenesis of HFpEF is basically related to mitochondrial damage through several interrelated mechanisms. Imbalance in energy substrate metabolism is a initiating factor for mitochondrial damage or dysfunction. This is manifested as impaired fatty acid oxidation and interrupted glucose utilization, resulting in persistent myocardial energy deficiency. Concurrently, dysregulation of mitochondrial calcium handling significantly contributes to disease progression. An increase in mitochondrial calcium concentration may initially maintain Adenosine-5’-triphosphate (ATP) production through compensatory mechanisms. However, sustained calcium overload eventually triggers the opening of the mitochondrial permeability transition pore, leading to increased oxidative stress and cellular apoptosis. Furthermore, abnormalities in mitochondrial dynamics, particularly excessive activation of dynamin-related protein 1 (Drp1), drive pathological mitochondrial fragmentation. This fission-fusion imbalance reduces ATP generation capacity, impairs mitophagy, and elevates oxidative stress, collectively worsening cardiac remodeling and diastolic impairment.^[3]^ These processes create a self-perpetuating vicious cycle, eventually leading to characteristic diastolic dysfunction and HFpEF myocardial sclerosis (Figure 1).

## The dual role of exercise in HFpEF management

The impact of exercise on mitochondrial structure and function is closely related to the intensity of exercise. Therefore, in the future therapeutic treatment of HFpEF, exercise may serve as a “double-edged sword”. This review suggests that moderate exercise may have surprising effects on the treatment of HFpEF, typically corresponding to 40%-60% of peak VO_2max_, exercise serves as a powerful physiological stimulus for mitochondrial adaptation.^[4]^ It enhances mitochondrial biogenesis primarily through activation of the PGC-1α/NRF1/TFAM signaling axis, leading to increased synthesis of oxidative phosphorylation enzymes and improved coupling efficiency between substrate oxidation and ATP synthesis. Moderate training also optimizes myocardial substrate utilization by reducing pathological dependence on fatty acid oxidation while significantly increasing glucose oxidation. In contrast, excessive or unaccustomed high-intensity exercise, particularly above 70% of peak VO_2max_, may overwhelm mitochondrial respiratory capacity. The relationship between exercise load and mitochondrial health thus follows a non-linear “dose-response” pattern, where moderate exercise promotes adaptation and resilience, while extreme or prolonged exertion may trigger maladaptive responses that exacerbate the underlying pathology of HFpEF.

## Exercise regulates multiple other pathways to improve HFpEF

Enhancing the endogenous antioxidant defense system is an important adaptive mechanism for exercise to improve mitochondria. Long-term exercise training upregulates mitochondrial antioxidants, such as manganese superoxide dismutase (MnSOD), directly reducing the production of hydrogen peroxide within mitochondria and establishing a strong defense barrier against oxidative stress caused by exercise.^[5]^

Neural activation of the nervous system after long-term exercise also has a potential impact on mitochondrial function. Research has found that the regulatory effect of a rich environment on the nervous system may extend to the cardiovascular mitochondrial system, exerting a beneficial influence on the pathological process of HFpEF. In addition, the social and cognitive stimuli provided by the environment may help regulate autonomic nerve balance and may alleviate the characteristic overactivation state of the sympathetic nerve in HFpEF.^[6]^

## Clinical translation and future perspectives

The clinical practice of treating HFpEF through exercise requires development in detection. The development of non-invasive technologies for monitoring mitochondrial function represents a promising direction for optimizing exercise prescriptions. Methods such as skin mitochondrial oxygen tension measurement offer the potential for repeated assessment during the intervention period, dynamically reflecting mitochondrial oxygen metabolism during the exercise and recovery phases. These measurements can establish a personalized “dose-response” relationship between exercise intensity and mitochondrial adaptation, helping to determine the optimal exercise threshold for each patient.

## Conclusion

Mitochondrial dysfunction is a key pathophysiological factor of HFpEF. Exercise, as a possible and effective non-pharmaceutical strategy, restores mitochondrial health through multiple complementary mechanisms. However, the therapeutic application of exercise in HFpEF requires careful calibration, as excessive intensity may overwhelm adaptability and potentially exacerbate underlying pathology. Future research should prioritize the development of precise, mitochondrial-targeted exercise interventions, combined with personalized threshold measurement and standardized monitoring protocols, to maximize benefits while ensuring the safety of HFpEF patients within the range of disease severity and comorbidities burden.
